# FXR-ApoC2 pathway activates UCP1-mediated thermogenesis by promoting the browning of white adipose tissues

**DOI:** 10.1016/j.jbc.2025.108181

**Published:** 2025-01-10

**Authors:** Sang Hee Kim, Woo Yong Park, Beomsu Kim, Jin-Hyung Kim, Gahee Song, Ja Yeon Park, Wenjun Jiao, Se Jin Jung, Kwang Seok Ahn, Hyun Jeong Kwak, Jae-Young Um

**Affiliations:** 1 Department of Science in Korean Medicine, Graduate School, Kyung Hee University, Seoul, Republic of Korea; 2 Department of Pharmacology, College of Korean Medicine, Kyung Hee University, Seoul, Republic of Korea; 3 Department of Biomedical and Pharmaceutical Science, College of Pharmacy, Kyung Hee University, Seoul, Republic of Korea; 4 Kyung Hee Institute of Convergence Korean Medicine, Kyung Hee University, Seoul, Republic of Korea; 5 Department of Bio and Fermentation Convergence Technology, Kookmin University, Seoul Republic of Korea

**Keywords:** FXR, ApoC2, UCP1, PGC1α, beige adipocyte

## Abstract

FXR, encoded by *Nh1r4*, is a nuclear receptor crucial in regulating bile acid, lipid, and glucose metabolism. Prior research has indicated that activating FXR in the liver and small intestine may offer protection against obesity and metabolic diseases. This study demonstrates the essential role of the FXR-ApoC2 pathway in promoting the browning of white adipose tissue (WAT). Increased FXR by treatment with the FXR agonist farnesol upregulated beige adipocyte markers, including UCP1, PGC1α, and PRDM16, and increased the FXR target gene, ApoC2, in beige adipocytes and cold-exposed mice. However, these effects were not observed in mature white adipocytes. Remarkably, the knockdown of FXR results in a significantly reduced expression of UCP1, PGC1α, PRDM16, and ApoC2 in beige adipocytes. While studying the interaction between the nuclear receptor RXRα and FXR in transcription regulation, it was found that the knockdown of RXRα did not control the expression of FXR under beige adipogenesis. We further investigated whether the expression of beige-related markers could be altered under ApoC2 overexpression to ascertain the mechanism of action of FXR in relation to ApoC2 regulation. The overexpression of ApoC2 in both preadipocytes and beige adipocytes led to a significant increase in the expression of UCP1 and PGC1α. These results indicate that the FXR-mediated ApoC2 pathway is essential in the browning of WAT by inducing beige adipogenesis from preadipocytes.

Adipose tissue is essential for energy homeostasis, as it functions as an energy reservoir and regulates energy balance through various mechanisms (1). It stores excess energy as fat, which can be mobilized during periods of energy deficiency (2). Additionally, it secretes hormones such as leptin and adiponectin, which communicate with the brain and other organs to regulate appetite, energy expenditure, and metabolic processes (3). This dynamic interaction contributes to the preservation of body weight and energy balance (4). Recent studies have highlighted the potential therapeutic benefits of activating beige adipocytes to protect against obesity and related diseases (5). These cells contribute to energy expenditure and are located in inguinal white adipose tissue (iWAT), where they can be stimulated by environmental factors including cold exposure, β3-adrenergic receptor agonists, and exercise (6). In contrast to classical white adipocytes, beige adipocytes, express elevated levels of uncoupling protein 1 (UCP1) (7). This protein enables white adipocytes to convert stored energy into heat, thereby increasing their energy expenditure (8). Despite the potential to alleviate obesity, beige adipocytes have significant limitations and challenges that require further investigation to ensure the safety and efficacy of the therapy (9).

The farnesoid X receptor (NR1H4, FXR) is a nuclear receptor that has a significant role in lipid homeostasis by regulating genes involved in lipid uptake, synthesis, and breakdown (10). FXR function is primarily highlighted as a novel therapeutic target for nonalcoholic steatosis (NASH), also known as metabolic dysfunction-associated steatohepatitis (MASH). Many FXR agonists have been developed for NASH/MASH therapy. Obeticholic acid (OCA) is the pioneering frontrunner FXR agonist and the first to demonstrate success in clinical trials (11). In addition, Fex, the gut-restricted FXR agonist, was shown to induce enteric fibroblast growth factor 15 (FGF15), leading to alterations in bile acid composition and promoting adipose tissue browning (12). Another FXR agonist, farnesol (FA), prevents hepatic lipid accumulation in rodents and humans by upregulating the farnesoid X receptor (13, 14, 15). FA is an endogenously created non-sterol isoprenoid derived from the dephosphorylation of farnesyl pyrophosphate (FPP), which is an intermediate in the HMG-CoA reductase pathway to make cholesterol and other isoprenoids out of acetyl-CoA in both plants and animals (16). In the context of adipose tissues, we previously reported that activation of adipose FXR by FA supplementation suppresses adipose tissue expansion by downregulating peroxisome proliferator-activated receptor γ (PPARγ) and increasing adipose tissue browning through the upregulation of AMPK phosphorylation in brown adipose tissue (BAT) and inguinal white adipose tissue (iWAT) of high-fat diet (HFD)-induced obese mice (17, 18). While it has been uncovered that adipose FXR activation modulates adipose tissue browning, the mechanism by which the transcriptional machinery drives beige adipocyte development and activation is poorly understood.

Apolipoprotein C2 (ApoC2) is essential for lipid metabolism because it is responsible for activating lipoprotein lipase (LPL), which has a fundamental role in breaking down triglycerides and very low-density lipoproteins (VLDL) into free fatty acids and glycerol (19). It has been demonstrated that *Apoc2*^−/−^ mice develop metabolic defects including hypertriglyceridemia and acute recurrent pancreatitis (20). Patients with a genetic deficiency of the ApoC2 gene exhibit hypertriglyceridemia (21, 22, 23). Moreover, a homozygous point mutation in the ApoC2 gene, resulting in no measurable plasma ApoC2 in infancy, causes severe hyperchylomicronemia and encephalopathy (24). Previous studies have demonstrated that FXR activation induces hepatic ApoC2, which activates LPL, while apolipoprotein C3 (ApoC3) expression is reduced, inhibiting LPL (25, 26). Moreover, adipocytes regulate extracellular LPL activity by expressing their own ApoC2 and ApoC3 (27), by which increased ApoC2 by FXR could potentially provide fatty acids that can serve as substrates for thermogenesis in beige adipocytes. However, research on the mechanisms by which the ApoC2 influences thermogenic adipocyte development and function is currently unclear. Here, we sought to investigate the transcriptional machinery of FXR-ApoC2 signaling in adipose tissue browning.

## Results

### FXR expression is elevated in the inguinal WAT, which undergoes browning after acute cold exposure

**Figure 1:**
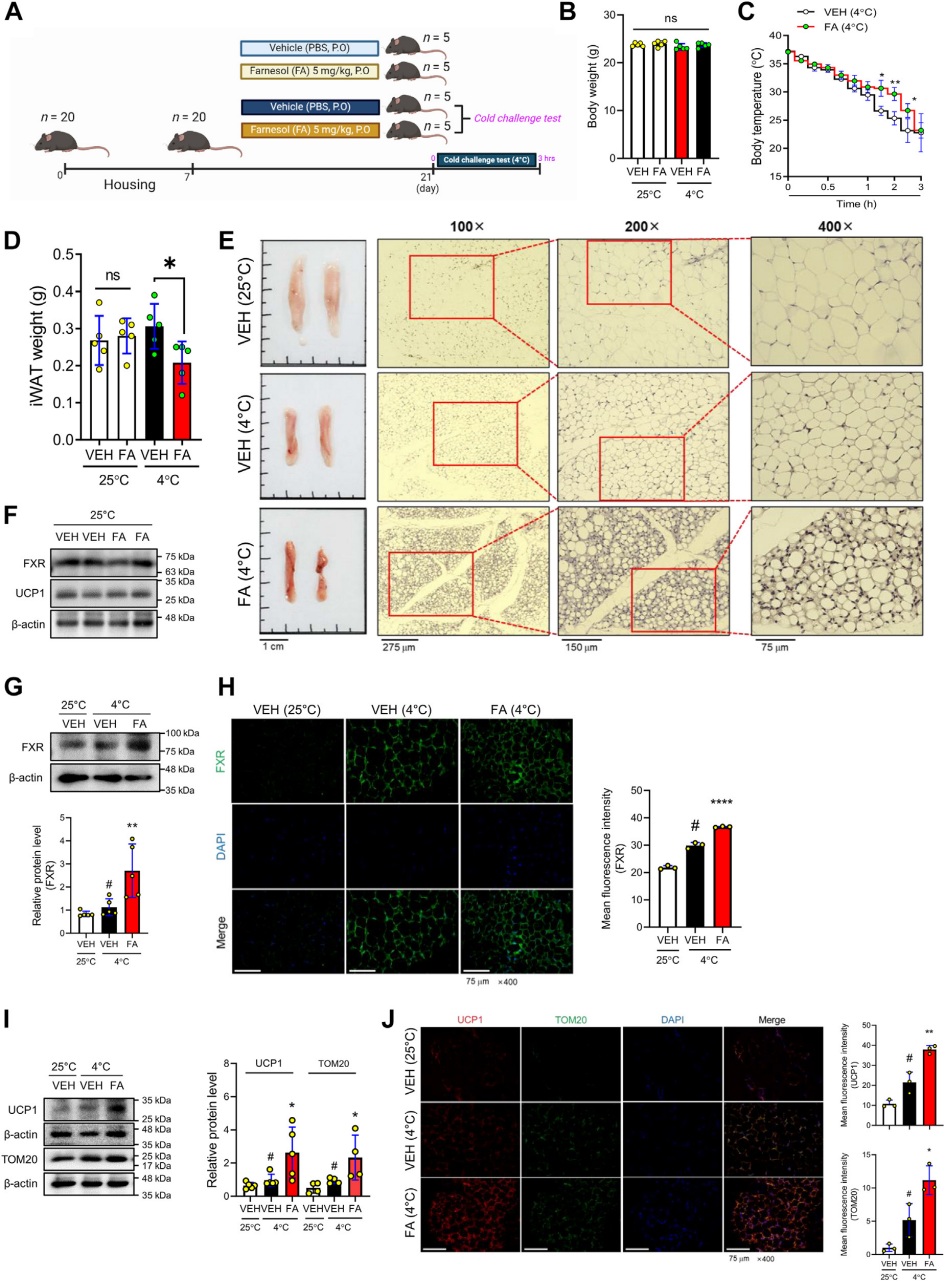
**Activation of FXR expression induces browning in the iWAT of cold-exposed C57BL/6J mice.***A*, experimental scheme of the *in vivo* study. Mice (*n* = 20) were randomly divided into four groups: vehicle group (PBS-fed) and farnesol group (farnesol-fed 5 mg/kg/day) exposed to cold or not. All groups were orally administered PBS or farnesol for 14 days, and the cold group was exposed for 3 h in a cold room at 4 °C. *B*, the body weight of the mice was measured. *C*, rectal temperature was measured at the indicated time points in mice kept at 4 °C for 3 h. *D*, weight of iWAT in PBS-/FA (5 mg/kg/day)-fed and exposed to 4 °C (*n* = 5 per group) mice was measured. *E*, representative images of the iWAT are shown and paraffin-embedded iWAT was stained with H&E (magnification × 100, × 200, × 400, scale bar = 275 μm, 150 μm, 75 μm). *F* and *G*, protein levels of FXR and UCP1 in the iWAT were analyzed with Western blot analysis. *H*, expression of FXR (*green*) and nuclei (*blue*) of the iWAT was evaluated by immunofluorescence staining (magnification × 400, scale bar = 75 μm). *I*, protein levels of UCP1 and TOM20 were measured in the iWAT with Western blot analysis. *J*, expression of UCP1 (*red*), TOM20 (*green*), and nuclei (*blue*) were detected in the iWAT by immunofluorescence staining (magnification × 400, scale bar = 75 μm). Quantification of the fluorescence intensities was determined using ImageJ. β-actin was used as a loading control. All data are expressed as the mean ± SD of three or more independent experiments. Statistical differences were evaluated using an unpaired *t* test and a subsequent *post hoc* one-tailed Mann-Whitney *U* test. ^#^*p* < 0.05 *versus* VEH group; ∗*p* < 0.05 *versus* VEH group exposed to cold; ∗∗*p* < 0.01 *versus* VEH group exposed to cold, iWAT, inguinal white adipose tissue; ns, non-significant; FA, farnesol.

To induce the browning of WAT, we used a test that included acute exposure to low temperatures (4 °C), as shown in Fig. 1*A*. The body weight of the mice was not altered after the cold challenge test (Fig. 1*B*). The body temperature of the mice, representing the rectal temperature, was obtained at the indicated time points in Fig. 1*C*. The mice exposed to the cold treatment exhibited an increased thermogenic capacity. We collected the iWAT to observe histological and physiological changes after the cold challenge. We used epidydimal WAT (eWAT) as the negative control because it does not undergo browning with acute cold challenge (28). The weight of the iWAT and size of the adipocytes in the iWAT in the FA (4 °C) mice were significantly lower compared to the VEH (4 °C) mice. Additionally, an obvious difference was observed in the iWAT between the VEH (4 °C) and FA (4 °C) mice (Fig. 1, *D* and *E*). Interestingly, the FXR mRNA and protein expression were increased in the iWAT of the FA (4 °C) mice but were not altered in the iWAT (25 °C) and eWAT (4 °C) of the FA mice (Fig. 1, *F* and *G* and Fig. S1, *A* and *B*). By IF staining, increased expression of FXR was observed in the iWAT of the FA (4 °C) mice compared to the VEH (4 °C) mice (Fig. 1*H*). It seems to upregulate the transcription rate of the FXR gene depending on the cold exposure. Studies have shown that FA supplementation upregulated the FXR protein expression in various tissues. The mRNA and protein expression of UCP1 was increased, and the protein level of TOM20, representing the mitochondrial abundance, was increased in the iWAT of the FA (4 °C) mice compared to the VEH (4 °C) mice in FA mice (Fig. 1*I*, Fig. S1*B*). Results from the IF staining with UCP1 and TOM20 in the iWAT validated the browning of iWAT in the FA (4 °C) mice (Fig. 1*J*). In general, the cold-induced browning process of WAT is stimulated by activating beta-3 adrenergic receptor (β3AR) signaling. In the iWAT of the FA (4 °C) mice, the protein expression of β3AR was increased, while the sequential protein expression of the cAMP-response element binding protein (CREB) was not altered (Fig. S1*C*). Meanwhile, it is also well known that FXR acts as a nuclear receptor to interact with multiple PPAR isotypes and is the master regulator for fatty acid and lipid metabolism-related genes in the liver, intestine, and adipose tissues. To validate that, the protein expression of three PPAR isotypes (PPARα, PPARδ, and PPARγ) was examined in the iWAT and it was increased in the FA (4 °C) mice (Fig. S1*D*). Overall, our results suggest that increased FXR expression by treatment with FA increased the thermogenic capacity in iWAT depots.

### FXR deficiency disrupts beige differentiation in preadipocytes

**Figure 2:**
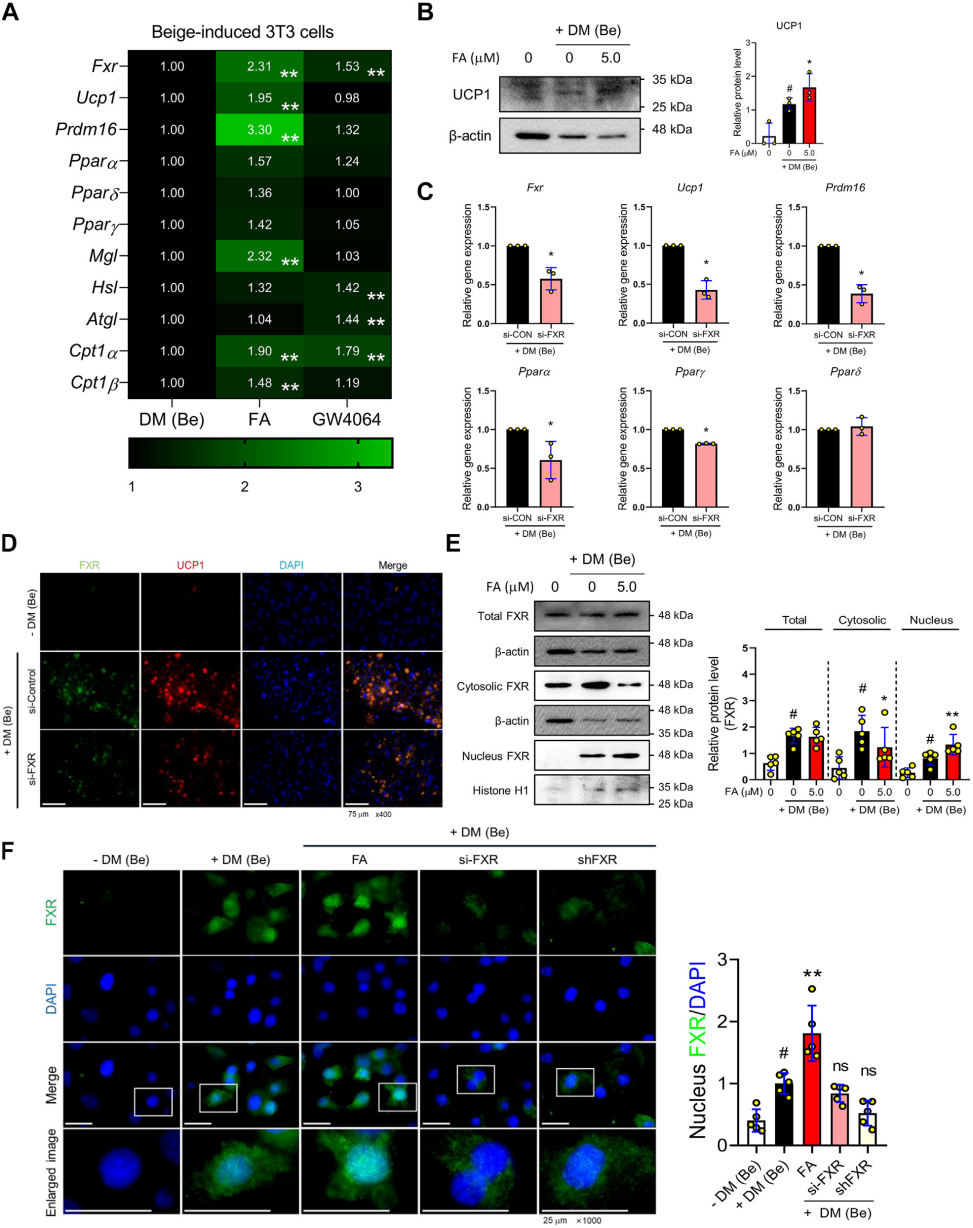
**Increased FXR translocation by farnesol induces beige-differentiation in 3T3-L1 cells.***A*, the heatmap of expression levels of 11 genes (*Fxr*, *Ucp1*, *Prdm16*, *Pparα*, *Pparδ*, *Pparγ*, *Mgl*, *Hsl*, *Atgl*, *Cpt1α*, and *Cpt1β*) in beige-induced 3T3-L1 cells treated with farnesol or GW4064 by RT-PCR analysis. *B*, the protein level of UCP1 was measured by Western blot analysis. *C*, the mRNA expressions of *Fxr*, *Ucp1*, *Prdm16*, *Pparα*, *Pparγ*, and *Pparδ* are measured by RT-PCR in beige-induced 3T3-L1 cells treated with si-FXR or si-Control. Results were expressed relative to *Gapdh*. *D*, expressions of FXR (*green*), UCP1 (*red*), and nuclei (*blue*) were detected by immunofluorescence staining (magnification × 400, scale bar = 75 μm) in beige-induced 3T3-L1 cells treated with si-FXR or si-Control. *E*, protein levels of FXR in the total, cytosolic, and nuclear fractions of beige-induced 3T3-L1 cells treated with farnesol by Western blot analysis. FXR protein level normalized with β-actin and Histone H1 for cytosolic and nuclear fractions, respectively. The levels of protein were quantified using ImageJ. *F*, expressions of FXR (*green*) and nuclei (*blue*) were detected by immunofluorescence staining (magnification × 1000, scale bar = 25 μm) in beige-induced 3T3-L1 cells treated with FA, shFXR, and si-FXR. Quantification of fluorescence intensities for colocalization between FXR and nuclei was determined using ImageJ. All data are expressed as the mean ± SD of three or more independent experiments. Statistical differences were evaluated using an unpaired *t* test and a subsequent *post hoc* one-tailed Mann-Whitney *U* test. ^#^*p* < 0.05 *versus* DM (Be)-untreated 3T3-L1 cells; ∗*p* < 0.05 *versus* DM (Be)-stimulated 3T3-L1 cells. ^*∗∗*^*p* < 0.01 *versus* DM (Be)-stimulated 3T3-L1 cells. DM (Be), Beige adipocyte differentiation media; ns, non-significant; FA, farnesol.

To validate the role of FXR on adipocyte browning, we used 3T3-L1 preadipocytes and induced differentiation into beige adipocytes with FA or GW4064, a small-molecule FXR agonist. Using 3T3-L1 cells, we conducted a time-tracking assay to observe the protein levels of beige markers and FXR, establishing an experimental beige adipogenesis model. Genetic markers for beige adipocytes, such as UCP1, T-box transcription factor 1 (TBX1), and FXR, were observed to be expressed during the maintenance stage (48–96h) (Fig. S2*A*). Therefore, we treated the cells with the agonists FA or GW4064 in the maintenance stage. The heatmap shows the mRNA expression obtained from the beige adipocytes treated with FA or GW4064 and the original graphs are expressed in Fig. S2*B*. The two different FXR agonists improved the FXR expression efficiently, although the FA treatment effectively increased the mRNA expression in the differentiation and function of beige adipocytes compared to GW4064 (Fig. 2*A*). Furthermore, we found that GW4064 treatment induced a high expression of lipolysis-related genes but had no effect on the gene expression of UCP1 and PRDM16 compared to FA. GW4064 has been found to enhance the expression of FXR by directly binding to it, although the precise mechanism of action of FA is still not fully understood. We hypothesized that the regulatory actions of the two agonists on FXR would have distinct impacts on beige differentiation. To examine that, we performed the molecular docking assays between the two different FXR agonists and FXR. As mentioned, GW4064 can potentially bind to FXR, whereas FA does not have the ability to bind to FXR. This suggests that FA could enhance FXR activity by collaborating with endogenous ligands such as chenodeoxycholic acid or by assisting the generation of ligands, given that chenodeoxycholic acid has a stronger binding affinity to FXR than FA (10). Furthermore, FA did not exhibit a binding affinity with UCP1 (Fig. S3, *A–D*). This data suggested that FA indirectly activates FXR and UCP1 expression. The treatment of FA significantly increased UCP1 protein expression in beige adipocytes (Fig. 2*B*). The cells were transfected with siRNA targeting *Nr1h4* (FXR) and underwent beige differentiation to investigate the transcriptional role of FXR on beige-related genes. It has been confirmed that the suppression of FXR leads to a decrease in the expression of beige-related genes, such as UCP1, PRDM16, and PPARs (Fig. 2*C*). These results were confirmed through IF staining experiments, revealing a decrease in UCP1 and FXR protein expression in the same cell model (Fig. 2*D*). CD137 protein expression, another genetic marker of beige adipocytes, decreased in cells transfected with *Nr1h4* siRNA. Interestingly, IF staining for FXR and CD137 indicated that their protein expression was reduced by FXR genetic inhibition (Fig. S4*A*). To verify this, the protein expression of the FXR was validated in cell lysates divided into cytoplasmic and nuclear fractions. It was shown that the protein level of FXR was localized in the nucleus throughout the beige differentiation process. Furthermore, the presence of FA enhanced this effect (Fig. 2*E*). To visualize the nuclear localization of FXR, IF staining with FXR was performed in the FA-treated or *Nr1h4* siRNA or *Nr1h4* shRNA-transfected cells. During beige differentiation, FXR nuclear localization was significantly increased by FA, whereas inhibiting FXR expression by siRNA or shRNA restricted FXR expression to the cytoplasm. (Fig. 2*F*). Recent research results have suggested the possibility of converting mature white adipocytes into beige adipocytes in the browning process of WAT. To determine the function of FXR in this context, we differentiated 3T3-L1 cells into mature adipocytes. Then, we treated them with two different agonists to confirm the gene expression of beige-related markers. Our results showed that an increase in the activity of FXR does not contribute to the trans-differentiation of white adipocytes into beige adipocytes (Fig. S5*A*). Overall, our findings indicate that FXR activates the expression of UCP1 and beige-related genes, suggesting that FXR is required for beige differentiation and function derived from preadipocytes.

### FXR binds to DNA as a monomer and enhances the expression of the FXR target gene ApoC2 during beige differentiation

**Figure 3:**
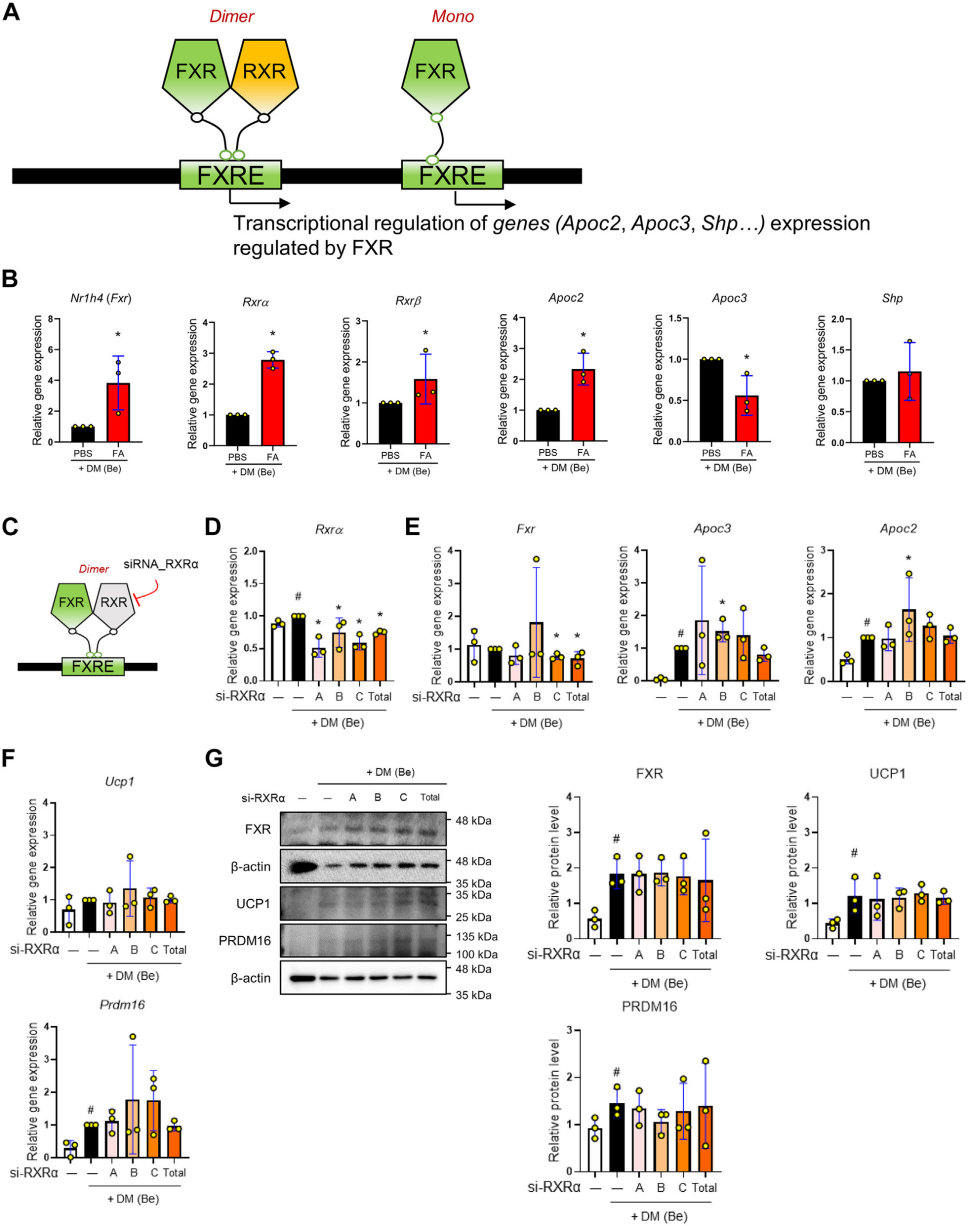
**FXR regulates gene transcription as a monomer in 3T3-L1 cells.***A*, schematic presentation of transcriptional mechanisms of gene expression regulated by FXR pathways. FXR binds to the FXR-response element (FXRE) as a heterodimer with retinoid X receptor (RXR) or as a monomer and regulates gene expression. *B*, the mRNA expressions of *Fxr*, *Rxrα*, *Rxrβ*, *Apoc2*, *Apoc3*, and *Shp* were measured by RT-PCR in beige-induced 3T3-L1 cells treated with farnesol. *C*, experimental design to confirm whether FXR regulates the gene as a heterodimer with RXRα. Beige-induced 3T3-L1 cells were treated with siRNA- RXRα. *D–F*, the mRNA expressions of *Rxrα*, *Fxr*, *Apoc3*, *Apoc2*, *Ucp1*, and *Prdm16* were measured by RT-PCR in beige-induced 3T3-L1 cells treated with si-RXRα A-C isotypes or si-Control (20 nM). Total indicated three isotypes combination treatment as final concentration of 20 nM. Results are expressed relative to *Gapdh*. *G*, UCP1, PRDM16, and FXR protein levels were measured in beige-induced 3T3-L1 cells treated with si-RXRα or si-Control by Western blot analysis. β-actin was used as a loading control. The levels of protein were quantified using ImageJ. All data are expressed as the mean ± SD of three or more independent experiments. Statistical differences were evaluated using an unpaired *t* test and a subsequent *post hoc* one-tailed Mann-Whitney *U* test. ^#^*p* < 0.05 *versus* DM (Be)-untreated 3T3-L1 cells; ∗*p* < 0.05 *versus* DM (Be)-stimulated 3T3-L1 cells. DM (Be), Beige adipocyte differentiation media; FA, farnesol.

It is well established that numerous genes could be regulated by FXR (Fig. 3*A*) (10). To validate the transcription activity of FXR, the expression of the FXR target genes *Apoc2*, *Apoc3*, *Shp,* and the two RXR subtypes, *Rxrα* and *Rxrβ*, was assessed in beige adipocytes. After FA treatment, the expression of *Nr1h4*, *Rxrα*, *Rxrβ*, and *Apoc2* was increased, while *Apoc3* mRNA expression was decreased. However, *Shp* gene expression remained unaffected (Fig. 3*B*). Our findings might suggest that activating the FXR-ApoC2 pathway during beige differentiation leads to increased lipids consumption by increasing the LPL activity. Research on the role of FXR has confirmed that it occurs alone or in dimerization with other kinds of nuclear receptors (10). Therefore, to further examine whether the thermogenic role of FXR involves dimerization with RXRs, we used the 3T3-L1 cells transfected with three different *Rxrα* siRNAs (Fig. 3*C*). The mRNA expression of *Rxrα* was diminished in all the cells transfected with *Rxrα* siRNA (Fig. 3*D*). The data showed that the suppression of RXR expression did not have an absolute regulatory effect on the mRNA expression of *Nr1h4*, *Apoc2*, and *Apoc3* (Fig. 3*E*). In addition, the gene expression in beige differentiation and function, including *Ucp1*, *Prdm16*, *Ppargc1α*, *Pparα*, and *Pparγ*, remained unaltered due to the inhibition of *Rxrα* expression (Fig. 3*F* and Fig. S4*B*). It was confirmed that FXR, UCP1, and PRDM16 protein levels were unaltered in the *Rxrα* siRNA-transfected and beige-differentiated cells (Fig. 3*G*). Based on these findings, it seems that the FXR-mediated thermogenic function did not depend on an interaction with RXRα.

### FXR-ApoC2 pathway contributes to beige differentiation in preadipocytes

**Figure 4:**
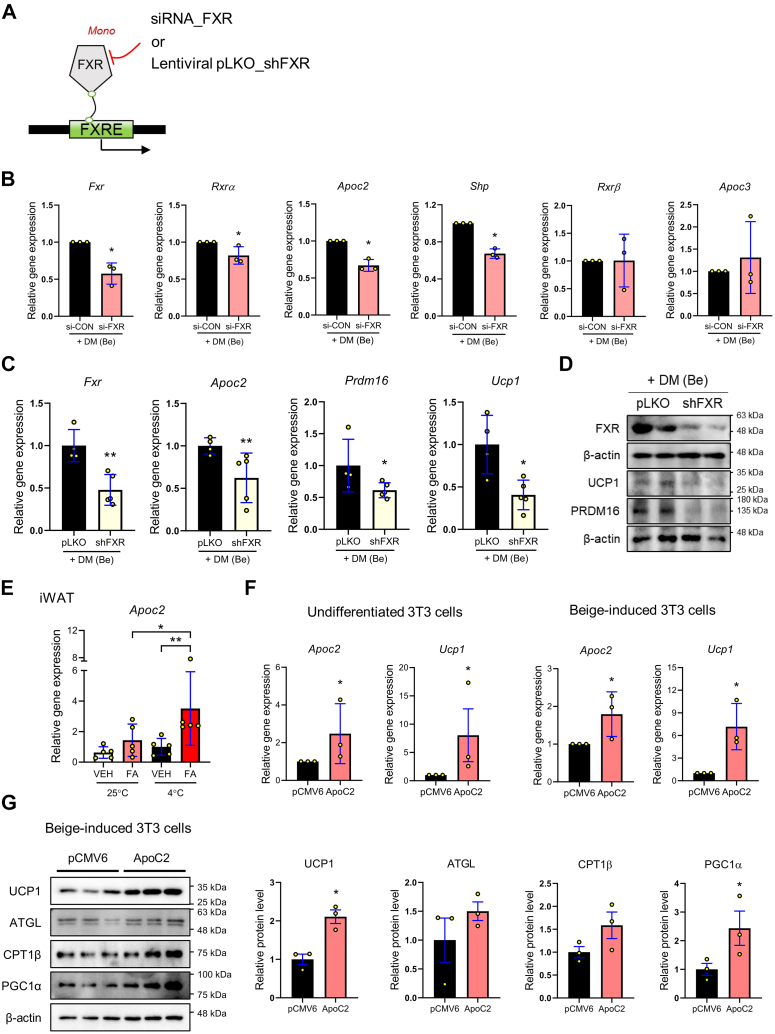
**FXR increases beige-differentiation regulating ApoC2 expression as a monomer in 3T3-L1 cells.***A*, experimental design to validate the regulation of genes by FXR in 3T3-L1 cells. Beige-induced 3T3-L1 cells were treated with siRNA- FXR and lentivirus pLKO encoding shFXR. *B*, the mRNA expressions of *Fxr*, *Rxrα*, *Rβ*, *Shp*, *Apoc2*, and *Apoc3* were measured by RT-PCR in beige-induced 3T3-L1 cells treated with si-FXR or si-Control. *C*, the mRNA expressions of *Fxr*, *Apoc2*, *Prdm16*, and *Ucp1* were measured by RT-PCR in beige-induced 3T3-L1 cells stably infected with lentivirus pLKO encoding shFXR or control shRNA. *D*, FXR, UCP1, and PRDM16 protein levels were measured in beige-induced 3T3-L1 cells transfected with lentivirus pLKO encoding shFXR or control shRNA. *E*, *Apoc2* mRNA expressions were determined in the iWAT of PBS-/FA (5 mg/kg/day)-fed mice exposed to 4 °C or not mice. *F*, the mRNA expressions of *Apoc2* and *Ucp1* were measured by RT-PCR in undifferentiated and beige-induced 3T3-L1 cells transfected with pCMV6 or ApoC2 expressing pCMV6. Results were expressed relative to *Gapdh*. *G*, UCP1, ATGL, CPT1β, and PGC1α protein levels were measured in beige-induced 3T3-L1 cells transfected with pCMV6 or ApoC2 expressing pCMV6 by Western blot analysis. β-actin was used as a loading control. The levels of protein were quantified using ImageJ. All data are expressed as the mean ± SD of three or more independent experiments. Statistical differences were evaluated using an unpaired *t* test and a subsequent *post hoc* one-tailed Mann-Whitney *U* test. Values of ∗*p* < 0.05 were considered statistically significant; iWAT, inguinal white adipose tissue.

**Figure 5:**
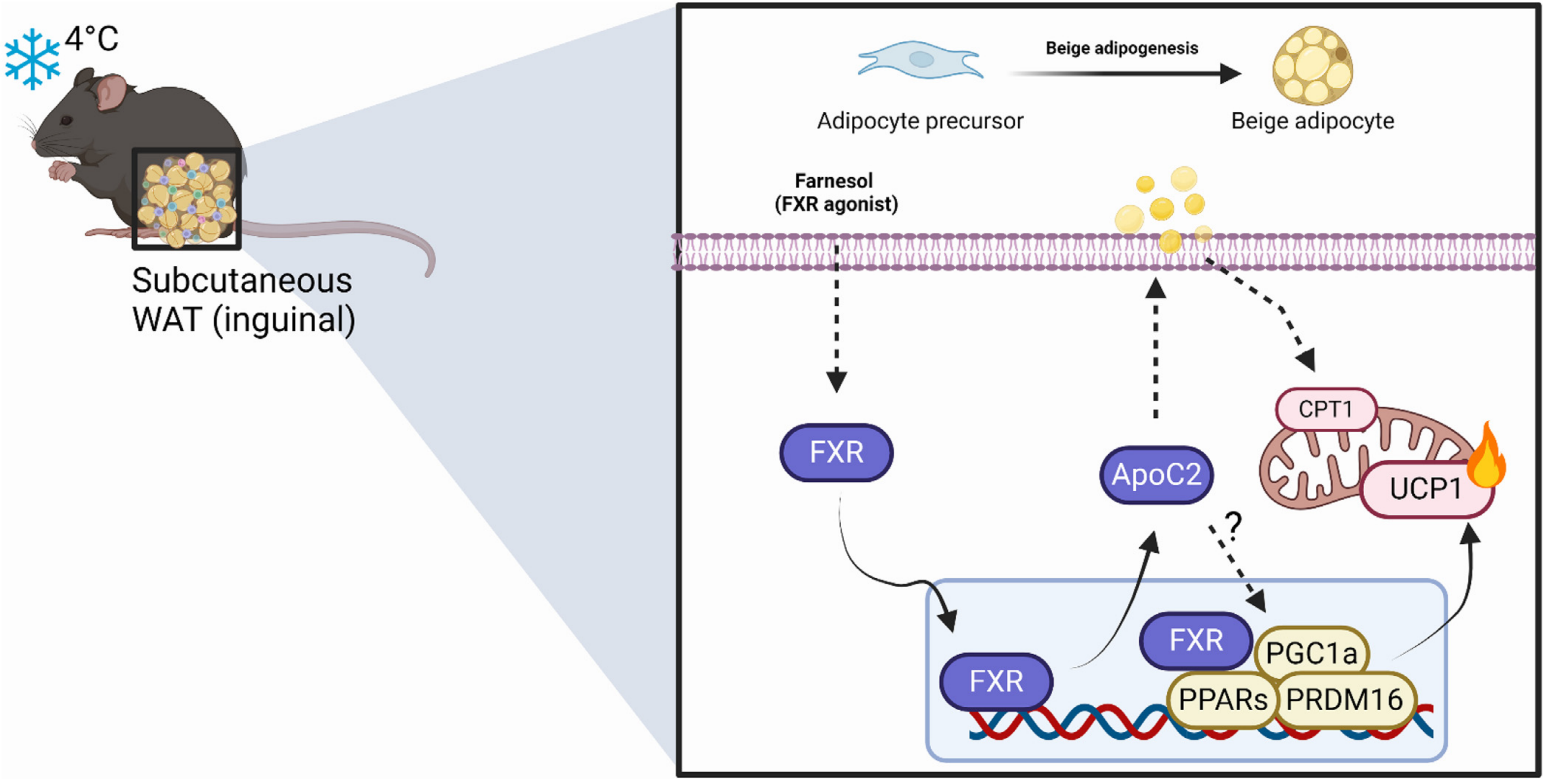
**Summary model.** Beige adipogenesis controls the increase in the number of mitochondria and the production of UCP1, a protein responsible for thermogenesis. This physiological alteration facilitates the manifestation of PPARs and PRDM16. The inhibition of the FXR-ApoC2 pathway suppressed beige adipogenesis, while ApoC2 overexpression promoted beige adipogenesis. The artwork was created on biorender.com.

Preadipocytes were treated with *Nr1h4* siRNA, and the gene expression of *Apoc2*, *Apoc3*, *Shp*, *Rxrα,* and *Rxrβ* were investigated (Fig. 4*A*) to understand better the role of FXR in controlling the elevated beige adipogenesis in the cells. The expression of the FXR target genes *Rxrα*, *Apoc2*, and *Shp* was decreased in the *Nr1h4* siRNA-transfected cells; however, the expressions of *Rxrβ* and *Apoc3* remained unaltered (Fig. 4*B*). Thus, the genetic deletion experiment was repeated with a lentiviral vector that included the *Nr1h4* shRNA to determine whether the FXR-ApoC2 pathway regulates beige differentiation in preadipocytes. The genetic deficiency of FXR resulted in a reduction in the mRNA expression of *Apoc2*, *Prdm16*, and *Ucp1*, which led to a decrease in the protein levels of beige differentiation factors such as PRDM16 and UCP1 (Fig. 4, *C* and *D*). Furthermore, we observed elevated *Apoc2* mRNA expression in the iWAT of the FA (4 °C) mice (Fig. 4*E*). Moreover, we investigated the potential correlation between ApoC2 expression and FA treatment. The molecular docking assay between ApoC2 and FA revealed that a strong binding potential was lacking between the two molecules (Fig. S3*E*). Thus, we conducted GFP-tagged *Apoc2* overexpression in preadipocytes to determine whether the ApoC2-dependent beige differentiation required FXR (Fig. S6*A*). Interestingly, ApoC2 overexpression significantly increased the *Ucp1* gene expression in both beige adipocytes and preadipocytes (Fig. 4*F*). Finally, it was demonstrated that the overexpression of ApoC2 leads to a significant increase in the protein levels of UCP1 and PGC1α (Fig. 4*G*) Taken together, these data suggest that FXR is required for beige adipogenesis; elevated ApoC2 expression *via* transcriptional activation of FXR enhances UCP1-mediated thermogenesis (Fig. 5).

## Discussion

This study shows that FXR-agonist FA-treated mice exhibit an increased cold tolerance and upregulated UCP1-mediated thermogenesis in the iWAT compared to PBS-fed mice. We validated this phenotype in beige-differentiated cells treated with FA using 3T3-L1 preadipocytes. The experiments with FXR or RXRα knockdown confirmed that the activator of the lipoprotein lipase ApoC2 is a strongly regulated gene in beige adipocytes. The results suggest that FXR might directly regulate ApoC2 without forming a dimer with RXRα. Furthermore, ApoC2 overexpression in beige adipocytes provides an advanced mechanism, explaining the improved beige differentiation and increased UCP1-mediated thermogenesis by FXR activation.

FXR activation was originally known for its regulatory function of lipid metabolism, particularly cholesterol metabolism, in various tissues such as the liver, intestine, and kidneys (29). It is a therapeutic target for treating human metabolic diseases. The role of FXR in adipose tissue, where it is moderately expressed, is not fully understood. In general, FXR is activated by bile acid (BA), which forms a binding collaboration with RXRs, triggering the activation of the SHP gene (30). This results in suppressing the key enzymes in cholesterol catabolism and primary BA synthesis (31). Numerous studies have demonstrated the significant induction of the SHP gene by various substances, including GW4064, androsterone, and chenodeoxycholic acid (32, 33, 34). FXR usually works in conjunction with RXRα and, when stimulated by bile acid, triggers the expression of SHP (35). Interestingly, our data showed that FA, a natural acyclic sesquiterpene alcohol, did not upregulate the expression of the SHP gene while increasing FXR and RXRα in beige adipocytes. Furthermore, the administration of FA led to a notable upregulation of genes that have a crucial role in beige differentiation, such as UCP1 and PRDM16. It could be proposed that the development of beige adipocytes is not dependent on the regulation of the SHP gene. In previous studies, it has been observed that RXR agonists have a positive effect on the differentiation of mesenchymal stem cells into adipocytes (36). However, during beige differentiation, we confirmed that genetic deletion of RXRα was insufficient to negatively regulate the expression of FXR and beige adipocyte-related markers. FXR activation might be believed to influence the browning of the iWAT when conducting the cold challenge. Our *in vitro* results suggest that FXR activation contributes to the differentiation of preadipocytes into beige adipocytes.

Moreover, FXR activation regulates the gene expression of ApoC2 and ApoC3 (36). Two hepatic regulatory regions (HCR.1 and HCR.2) have been shown to govern the liver-specific expression of the human ApoC2 gene cluster, containing components essential for enhancing ApoC2 expression in the liver. Kast *et al.* verified that FXR promotes ApoC2 transcription by directly binding the regions in HepG2 cells and substantiated this regulation in FXR K/O mice (26, 37, 38). ApoC2 in activating LPL is critical for the efficient breakdown of TG-rich lipoproteins in the bloodstream (39). Genetic deletion of ApoC2 impairs the ability of LPL to break down lipoproteins that are rich in triglycerides, resulting in a significant increase in plasma triglyceride levels and eventually causing severe hypertriglyceridemia and spontaneous atherosclerosis (40, 41). However, ApoC3 has a vital role as an inhibitor of LPL, resulting in a delay in lipid metabolism and the accumulation of lipid deposits. It has been reported that individuals with elevated plasma levels of ApoC3 have an elevated risk of experiencing cardiovascular diseases (42, 43). In the present study, we found that the cold-exposed mice fed the FA had an upregulated Apoc2 gene expression in the iWAT. Additionally, it was confirmed that activation of FXR by FA treatment was identified to increase ApoC2 and decrease ApoC3 gene expression in beige adipocytes.

We found that ApoC2 overexpression beige adipocytes induced UCP1 and PGC1α expression. Given the current findings, the precise relationship between ApoC2 and beige differentiation-related genes has yet to be defined. It is interesting to consider the probable mechanism by which the FXR-ApoC2 pathway may be necessary for beige adipogenesis. There are three potential scenarios that we can consider based on previous studies. First, the reason that FFAs are produced when ApoC2 activates LPL indicates that ApoC2 and UCP1 might be connected. These FFAs enhance the gene expression of UCP1 in BAT and WAT, promoting heat generation and thus contributing to thermoregulation and energy expenditure (44, 45). Second, recent research has shown that cyclic adenosine monophosphate (cAMP) could regulate the ApoC2 gene. Streicher *et al.* discovered a cAMP response element in the ApoC2 gene promoter and found that treatment with the cAMP activator forskolin increased the mRNA expression of ApoC2 in HepG2 cells (46). Our previous studies showed that activation of FXR by FA treatment in mice fed a HFD protected against body weight gain, induced iWAT browning as well as activated BAT by activating AMP-activated protein kinase (AMPK) phosphorylation (17, 18). In addition to the mouse model, we previously reported that FA treatment increased UCP1 and beige adipose markers in human adipose tissue-derived mesenchymal stem cells (17). It is well established that AMPK has an important role in developing and maintaining brown and beige adipose tissues (47). Third, activation of FXR can induce the expression of PGC1α, a crucial regulator of mitochondrial biogenesis and function. PGC1α has a vital role in forming thermogenic beige adipocytes by enhancing the expression of genes related to mitochondrial oxidative metabolism and thermogenesis (48). Our study revealed increased mRNA and protein levels of PGC1α by activating FXR in cells and animal models (Figs. 1 and 2). Furthermore, the genetic suppression of FXR by siRNA could reduce the expression of PGC1α in beige adipocytes (Fig. S6, *B* and *C*). Together, it is worth discussing arguments that not only support the fact that the FXR-ApoC2 pathway regulates the metabolism of lipoproteins but also show that it might be involved in developing beige adipocytes from preadipocytes.

In summary, our findings revealed a strong correlation between FXR-ApoC2 and the thermogenic program of WAT, which was further confirmed by suppressing FXR and overexpressing ApoC2 expression. Our research suggests that the FXR-ApoC2 pathway is crucial in the process of beige differentiation in preadipocytes. These findings enhance our understanding of the molecular mechanism of FXR in energy expenditure. Furthermore, these studies surprisingly uncover a new therapeutic strategy to manipulate adipose precursor to UCP1-positive cells without cold exposure through adipose precursor-based ApoC2 activation. Although FA supplementation would not increase FXR and UCP1 protein expression in iWAT of mice without cold exposure as well as in mature adipocytes, we found that overexpressing ApoC2 dependent or independent by FXR increases UCP1 mRNA expression in preadipocytes. The results suggest that adipose ApoC2 activation may offer an alternative strategy for controlling UCP1-mediated energy expenditure to combat metabolic disorders.

## Experimental procedures

### Chemical reagents

Dulbecco-modified eagle medium (DMEM), Penicillin-streptomycin, and fetal bovine serum (FBS) were purchased from Gibco. GW4064 was purchased from Tocris Bioscience (Bristol, UK). Sodium chloride was purchased from BioShop (ON, CA). Tris was purchased from Duchefa Biochemie (NH, NLD). Farnesol, 3-isobutylmethylxanthine (IBMX), dexamethasone, insulin, T3, rosiglitazone, and all other chemicals were purchased from Sigma Aldrich. Information on antibodies is provided in Table S1.

### Animal experiments

Male C57BL/6J mice (7-week-old, *n* = 20) were purchased from Deahan Biolink Co. and kept for 1 week prior to the experiments, then were randomly divided into 25 °C vehicle (*n* = 5), 4 °C vehicle (*n* = 5), 25 °C + FA group (*n* = 5), and 4 °C + FA group (*n* = 5). Mice were maintained at 22 to 25 °C throughout the 14 day normal diet (CJ Feed Co., Ltd, Seoul, South Korea) and FA (5 mg/kg) treatment period. After 14 days of treatment, for testing acute cold exposure challenge, mice were then placed at 4 °C, and body temperature was measured at 0.5, 1, 2, and 3 h. Body temperature was measured using a rectal probe connected to a testo 925 digital thermometer (Testo Inc., Lenzkirch, Germany). After euthanasia, inguinal white adipose tissue (iWAT) and epididymal white adipose tissue (eWAT) were collected and placed in −80 °C until further use. All animal experiments were performed according to the Guide for the Care and Use of Laboratory Animals and were approved by the Institutional Review Board of Kyung Hee University (confirmation number: KHUASP (SE)-13 to 012).

### Protein extraction and Western blot analysis

Protein extraction and Western blot analysis were performed as previously reported (17). The cells and tissues were lysed in cell lysis assay buffer (Sigma-Aldrich) on ice for 30 min. The whole lysates were resolved by sodium dodecyl sulfate (SDS)-polyacrylamide gel electrophoresis and transferred onto Polyvinylidene difluoride membranes (Merck KGaA). The membranes were blocked in 5% skim milk and incubated with primary antibodies (1:1000) overnight at 4 °C. Subsequently, they were incubated for 1h at room temperature with horseradish peroxidase-conjugated secondary antibodies (1:10,000). Protein signals were detected using the electrochemiluminescence advance kit (GE Healthcare Life Sciences). Quantification of the protein level was determined using ImageJ software (NIH, MD, USA).

### Subcellular fractionation

For cytosolic fractionation, cells were lysed in a cytosolic fraction buffer (150 mM sodium chloride, 50 mM Tris, 1% Triton, 0.5 mM phenylmethylsulfonyl fluoride, and pH 8) on ice for 5 min. Then, the nuclei were pelleted by centrifugation at 10,000 rpm for 7 min at 4 °C, and the cytosolic fraction (supernatant) was recovered. The pellet containing the nuclei was resuspended in cell lysis assay buffer (Sigma-Aldrich) on ice for 30 min and subsequently centrifuged at 13,000 rpm for 30 min at 4 °C to recover a nucleus fraction (supernatant). The purity of the fractions was assessed using Histone H1 as a nucleus marker and β-actin as a cytosolic marker.

### Hematoxylin and eosin staining

Hematoxylin and eosin (H&E) staining was performed as previously reported (49). Briefly, isolated tissues were collected and fixed in 10% formalin for 1 week and followed by embedded in paraffin. Tissue sections were deparaffinized in xylene, rehydrated with ethanol/water, and then stained with H&E. Microscopic examinations were conducted, and photographs were taken under an EVOSR Cell Imaging systems (Thermo Scientific).

### Cell culture and differentiation

The 3T3-L1 cells, a mouse embryo fibroblast cell line (American Type Culture Collection) were cultured and differentiated into beige adipocytes as previously described (50). The 3T3-L1 cells were cultured in DMEM plus 10% FBS and 100 U/ml of penicillin and streptomycin at 37 °C in an incubator with 5% CO_2_ until reaching 100% confluence. After 2 days of reaching full confluence (Day 0), the cells were differentiated with a differentiation medium (500 μM IBMX, 1 μM dexamethasone, 167 μM insulin, and 20 μM T3) and added to DMEM plus 10% FBS for 2 days (Day 2). Then, the medium was replaced by a maintenance medium (500 μM IBMX, 167 μM insulin, 20 μM T3, and 0.5 μM rosiglitazone) every 2 days (two times, Day 4–6). On Day 6, the cells were treated with farnesol or GW4064 for 2 days in the maintenance medium.

The HEK293T cells, a human embryonic kidney epithelial cell line (American Type Culture Collection), were used for lentivirus packing cells. The cells were cultured in DMEM plus 10% FBS and 100 U/ml of penicillin and streptomycin at 37 °C in an incubator with 5% CO_2_.

### Bacterial strains

*Escherichia coli* (*E.Coli*) strain DH5α was used for plasmid purification. DH5α was grown in Luria Bertani (LB) broth (BD Biosciences) supplemented with 100 μg/ml ampicillin (Biomax, Seoul, Korea) to select for pLKO.1-shFXR and scramble shRNA at 37 °C.

### Immunofluorescence (IF) staining

IF staining was performed as previously reported (50). The 3T3-L1 cells and tissues were fixed in 10% formalin and blocked with 5% bovine serum albumin (Calbiochem). Then, the cells and tissues were incubated with the indicated primary antibodies (1:100 in 5% bovine serum albumin) at 4 °C overnight. After their washing, the cells and tissues were incubated with Alexa Fluor 488- or 633-conjugated secondary antibody (1:1000) for 1 h. DAPI was used to stain cell nuclei. The fluorescence images were taken with a Fluoview FV1000 confocal microscope (Olympus) at the Core Facility for Supporting Analysis & Imaging of Biomedical Materials at Wonkwang University supported by the National Research Facilities and Equipment Center. The intensity of green or red in each image was quantified by using ImageJ software and visualized by GraphPad Prism version 8 (GraphPad Software).

### RNA extraction and real-time reverse transcription-polymerase chain reaction (RT-PCR)

RNA extraction and RT-PCR were performed as previously reported (50). Total RNA was isolated from 3T3-L1 cells and tissues using a GeneAll RiboEx total RNA extraction kit (GeneAll Biotechnology). Subsequently, cDNA synthesis was performed with a Maxime RT PreMix Kit (iNtRON Biotechnology) according to the manufacturer’s instructions. RT-PCR was performed using a SYBR Green Power Master Mix (Applied Biosystems) and a Step One Real-Time PCR System (Applied Biosystems). The mRNA expression of *Gapdh* was used as an endogenous control. Details of all primers are described in Table S2.

### si-RNA transfection

*si-FXR* (Rat si-RNA, Locus ID 60351, #4390771) (Invitrogen, CA, USA) and *si-RXRα* (Mouse si-RNA, Locus ID 20181, #SR415164) (OriGene Technologies Inc., Rockville, MD) were transfected into 3T3-L1 cells using Lipofectamine 2000 Reagent (Invitrogen) according to the manufacturer's instructions. A final concentration of 20 nM of siRNA was added to the media three times during cell culture and differentiation: 70% confluence of cells, on Day 0, and Day 2.

### Generation and isolation of *Fxr* knockdown plasmid vector

*Fxr* knockdown plasmid vectors were generated by restriction cloning according to the protocol available on the Addgene website [https://www.addgene.org/protocols/plko/]. The shRNA sequence for *Fxr* was cloned from the pLKO.1 (#10878, OriGene Technologies Inc.) using the flowing primers; forward primer: 5′-CCG GCG CCG TGT ACA AGT GTA AGA ACT CGA GTT CTT ACA CTT GTA CAC GGC GTT TTT G-3′, and reverse primer; 5′-AAT TCA AAA ACG CCG TGT ACA AGT GTA AGA ACT CGA GTT CTT ACA CTT GTA CAC GGC G-3’. These primers were designed with the Broad Institute's TRC shRNA Design Process and contain the shRNA sequence flanked by sequences compatible with the sticky ends of AgeI and EcoRI. pLKO.1 was digested with AgeI (#R0552S, New England Biolabs) and EcoRI (#R0101S, New England Biolabs). The resulting fragments were ligated at a 3:1 ratio of insert to vector using T4 DNA ligase (#M0202S, New England Biolabs) and T4 DNA ligase buffer (#B0202S, New England Biolabs).

The ligated plasmid was transformed into chemically competent DH5α *E.Coli*. Successful insertion of the pLKO.1-shFXR was confirmed by the growth in LB broth containing 100 μg/ml ampicillin (Biomax). According to the manufacturer's instructions, Plasmid DNA isolation was performed using an Exfection Plamid LE Midi kit (GeneAll Biotechnology). The plasmid DNA level was quantified using a Nanodrop 2000 Spectrophotometer (Thermo Fisher Scientific).

### Lentivirus packaging, and infection

HEK293 T cells were used for the transient transfections and production of recombinant retroviruses. When the packing cells reached 70% confluency, they were co-transfected with 250 ng pMD2.G (#12259, OriGene Technologies Inc), 750 ng psPAX2 (#12260, OriGene Technologies Inc), and 1 μg of either pLKO.1-shFXR or scramble shRNA (#1864, OriGene Technologies Inc) using the Lipofectamine 3000 Reagent (Invitrogen) according to the manufacturer's instructions. After 15 h of co-transfection, the medium was replaced with fresh medium for two consecutive days, during which the cell culture medium was collected. The collected medium was centrifuged at 1250 rpm for 5 min to remove cell debris and passed through a 0.45 μm filter. For viral infections, when the 3T3-L1 cells reached approximately 70% confluence, 1 ml of viral stock was directly added to the growth medium of the target cells and incubated at 37 °C in 5% CO_2_. After 24 h, the medium was replaced with fresh medium.

### Overexpression of ApoC2

For exogenous overexpression of *ApoC2*, when 3T3-L1 cells reached approximately 70% confluence, the cells were transfected with either 2.5 μg of pCMV6 (control vector; PS100010, OriGene Technologies Inc., MD, USA) or pCMV6-ApoC2 (pCMV6 containing the gene encoding *Apoc2*, MG200276, OriGene Technologies Inc) using the Lipofectamine 3000 Reagent (Invitrogen) according to the manufacturer's instructions. After 24 h post-transfection, the medium was replaced with fresh medium, and the cells were visually assessed for transfection efficiency by GFP fluorescence using an EVOSR Cell Imaging system (Thermo Scientific).

### Molecular docking calculations

The protein 3D crystal structure of FXR, UCP1, and ApoC2 were obtained from the RCSB PDB database (PDB ID: 6HL0, 8HBV, 1SOH, resolution: 1.66 Å, 2.51 Å). The ligands and water were removed to perform molecular docking, and hydrogen atoms were added. The 3D structures of the ligand, GW4064, and farnesol were minimized using the force field MMFF94 calculation in Chem3D Pro 14.0 (Cambridge Soft: PerkinElmer Inc). Molecular docking calculations were performed with AutoDock Vina and AutoDock Tools 1.5.6 (The Scripps Research Institute) utilizing the hybrid Lamarckian Genetic Algorithm (LGA). The grid box size was 60 Ǻ × 60 Ǻ × 60 Ǻ with 0.375 Ǻ. The 3D protein-ligand complex structure was selected with the lowest energy (RMSD < 1.0) and visualized by PyMOL 2.5.7 (Schrodinger LLC).

### Statistical analysis

All data are expressed as the mean ± SD of three or more independent experiments. Statistical difference was analyzed by one-tailed Student’s *t* test and a subsequent *post hoc* one-tailed Mann-Whitney *U* test using Prism 8 (GraphPad Software). Values with ∗*p* < 0.05, ∗∗*p* < 0.01, ∗∗∗*p* < 0.001, and ^*∗∗∗∗*^*p* < 0.0001 were considered statistical significance.

## Data availability

All data will be made available on request.

## Supporting information

This article contains supporting information.

## Conflict of interest

The authors declare that they have no conflicts of interest with the contents of this article.
