# Supplementary material for: FXR-ApoC2 pathway activates UCP1-mediated thermogenesis by promoting the browning of white adipose tissues
Source: J Biol Chem. 2025 Jan 10;301(3):108181. doi: 10.1016/j.jbc.2025.108181 (PMC11871442; doi:10.1016/j.jbc.2025.108181)
Supplement: Figures S1-S6 and Tables S1 and S2 [file mmc1.docx]

***Supporting information***

***FXR-ApoC2 pathway activates UCP1-mediated thermogenesis by promoting the browning of white adipose tissues***

*Supplementary Figure 1*

*Supplementary Figure 2*

*Supplementary Figure 3*

*Supplementary Figure 4*

*Supplementary Figure 5*

*Supplementary Figure 6*

*Supplementary Table 1*

*Supplementary Table 2****
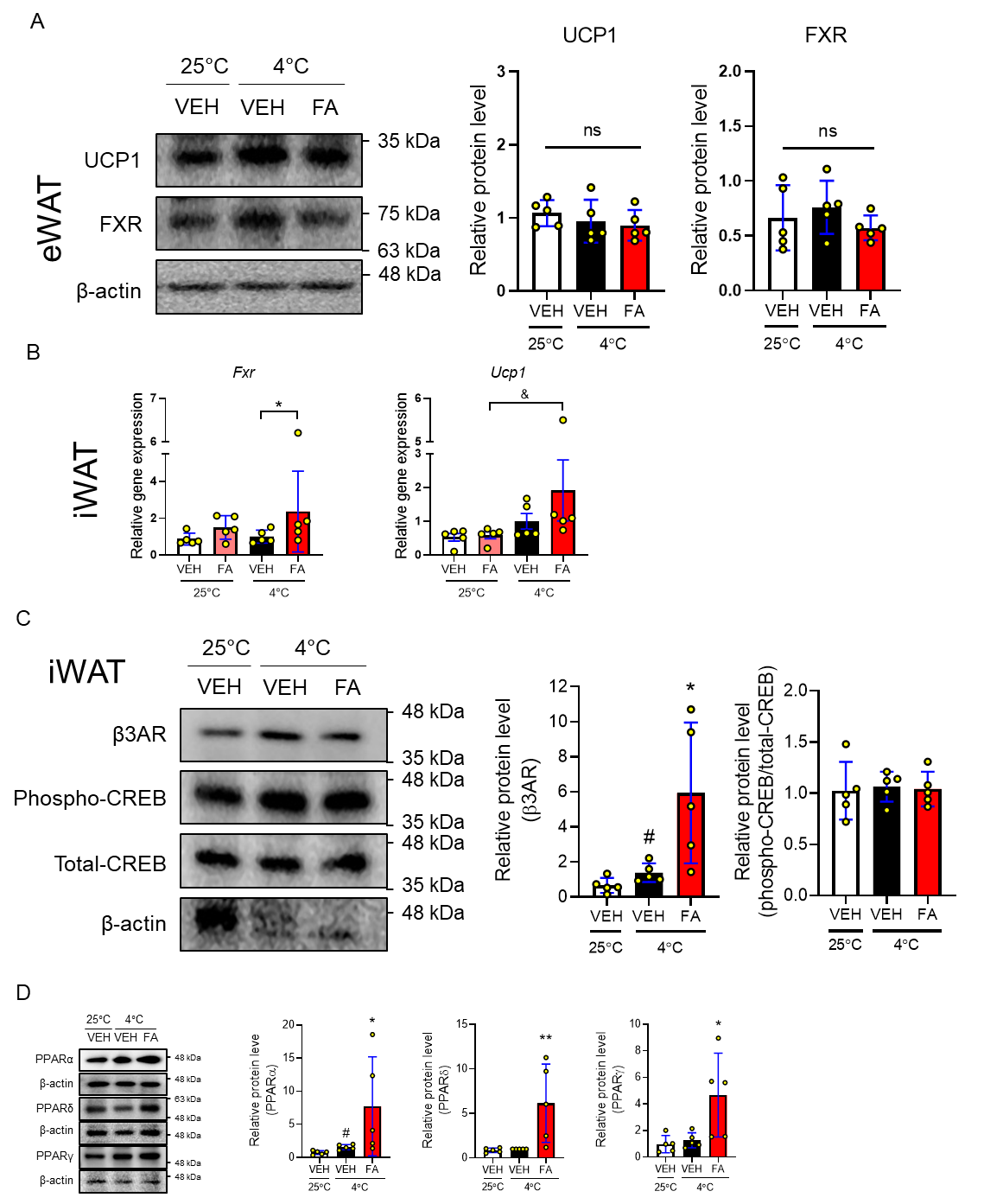
***

***Supplementary Figure 1***

(A) Protein levels of UCP1 and FXR were measured in the eWAT of PBS-/FA (5 mg/kg/day)-fed exposed to cold or not by western blot analysis. (B) The mRNA expression of *Fxr* and *Ucp*1 in the iWAT of PBS-/FA (5 mg/kg/day)-fed exposed to cold or not measured by RT-PCR analysis. Results were expressed relative to *Gapdh*. (C) Protein levels of β3AR, and phosphorylated CREB (Ser133) were measured. Phospho-CREB protein level was normalized to total CREB. (D) Protein levels of PPARα, PPARδ, and PPARγ in the iWAT were analyzed with western blot analysis. β-actin was used as a loading control. The levels of protein were quantified using ImageJ. All data are expressed as the mean ± SD of three or more independent experiments. Statistical differences were evaluated using an unpaired *t*-test and a subsequent *post hoc* one-tailed Mann-Whitney *U* test. ^#^*p* < 0.05 vs VEH group; **p* < 0.05 vs VEH group exposed to cold; ***p* < 0.01 vs VEH group exposed to cold; ^&^*p* < 0.05 vs FA group, iWAT, inguinal white adipose tissue; eWAT, epididymal white adipose tissue; ns, non-significant; FA, farnesol

***
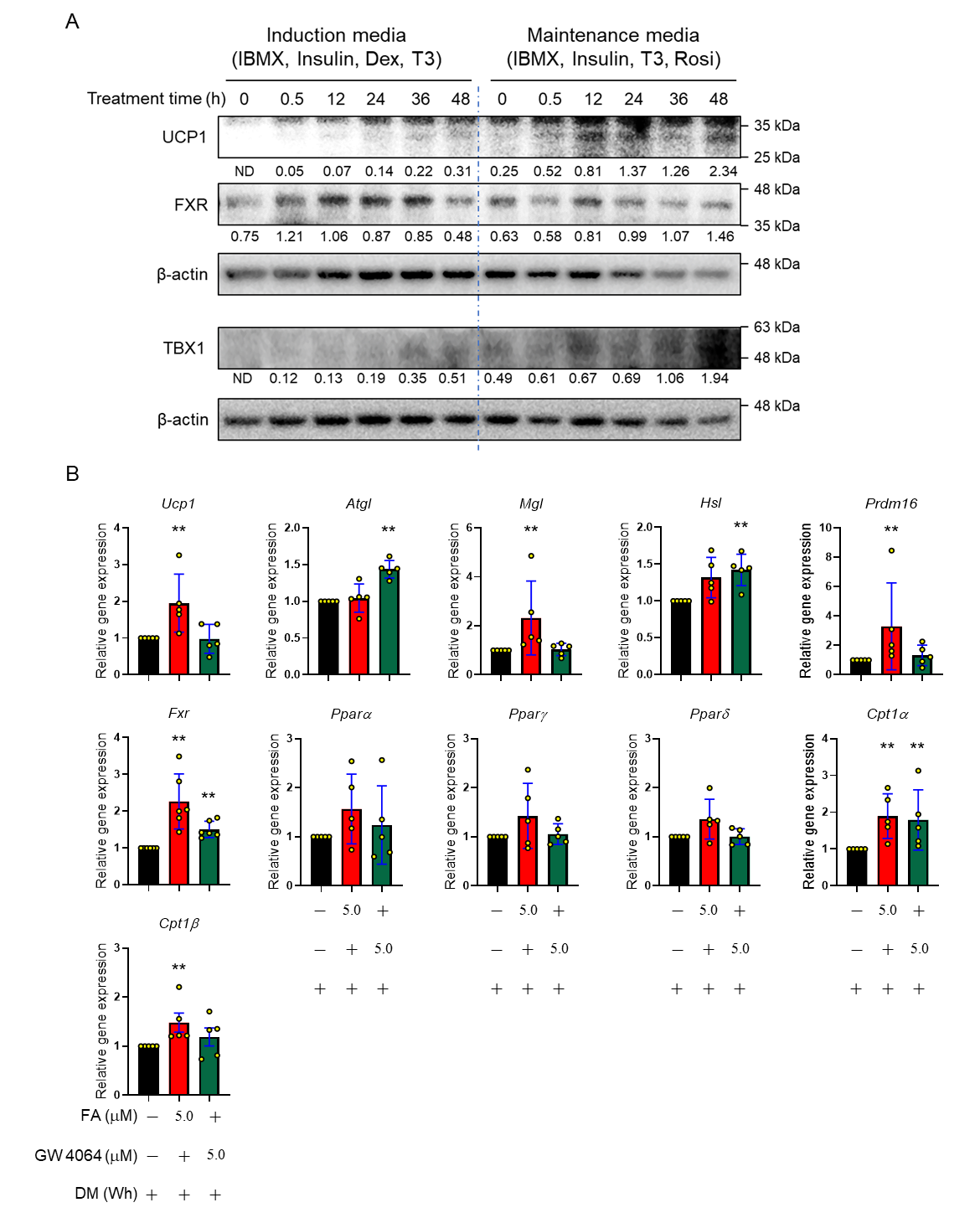
Supplementary Figure 2***

(A) Protein levels of UCP1, FXR, and TBX1 in 3T3-L1 during beige differentiation. β-actin was used as a loading control. The levels of protein were quantified using the ImageJ. (B) The mRNA expression of *Ucp1*, *Atgl*, *Mgl*, *Hsl*, *Prdm16*, *Fxr*, *Pparα*, *Pparγ*, *Pparδ*, *Cpt1α*, and *Cpt1β* in beige-induced 3T3-L1 cells treated with farnesol or GW4064 by RT-PCR analysis. Results were expressed relative to *Gapdh*. RT-PCR data are expressed as the mean ± SD of three or more independent experiments. Statistical differences were evaluated using an unpaired *t*-test and a subsequent *post hoc* one-tailed Mann-Whitney *U* test. *^**^p* < 0.01 vs DM (Be)-stimulated 3T3-L1 cells. DM (Be), Beige adipocyte differentiation media; FA, farnesol.


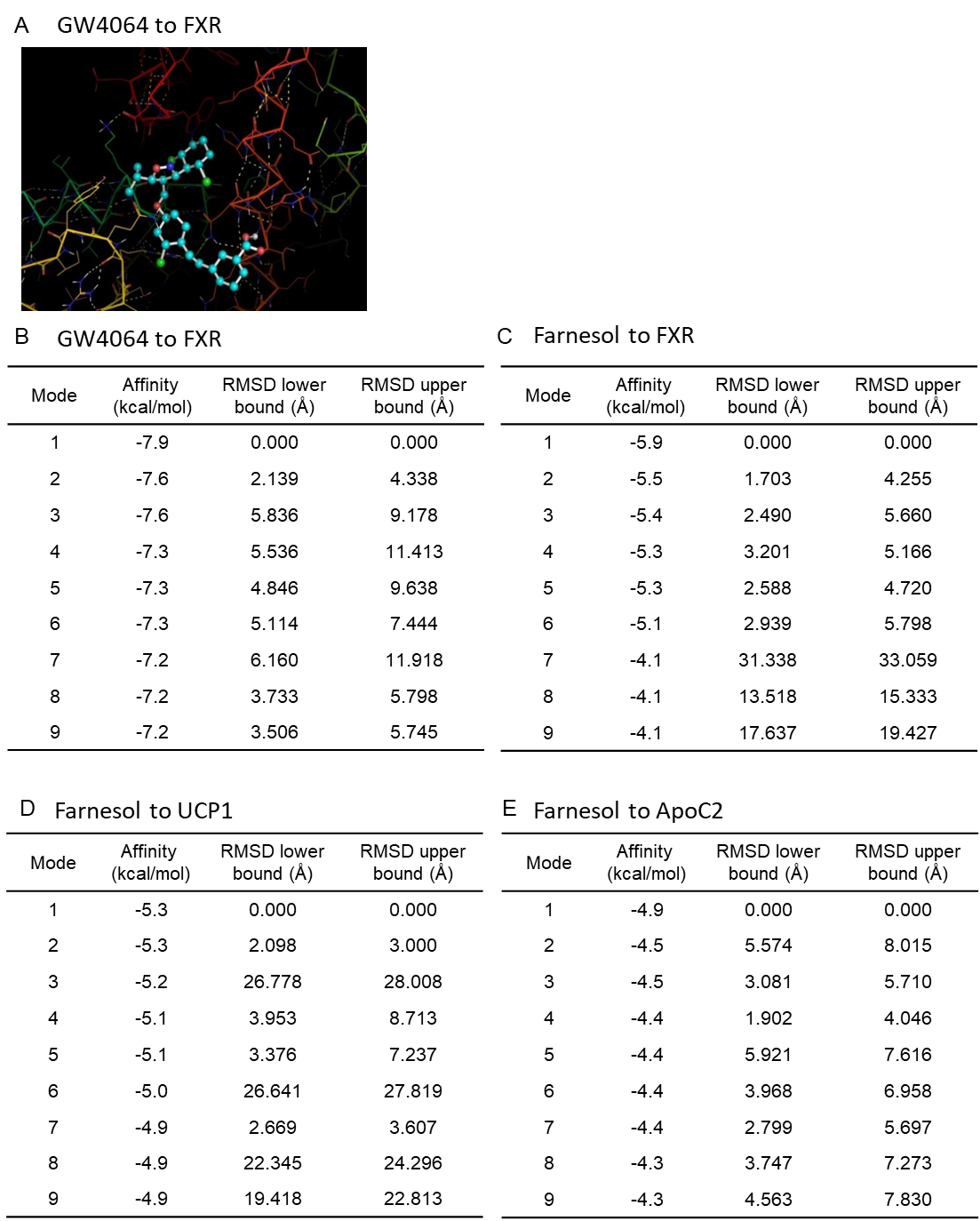


***Supplementary Figure 3***

(A) 3D structure of molecular docking between the protein FXR and GW4064. (B) Docking parameters of GW4064 to FXR. (C) Docking parameters of Farnesol to FXR. (D) Docking parameters of Farnesol to UCP1. (E) Docking parameters of Farnesol to ApoC2. All data are calculated using AutoDock Vina. The binding affinities (kcal/mol) were calculated for 9 docking sites. The lower value of Root-mean-square deviations of atomic positions (RMSD) means that docking is validated with higher accuracy. RMSD values of 3 or more indicate docking has not occurred. Mode 1, One docking position, with RMSD = 0, is highly valid.

***
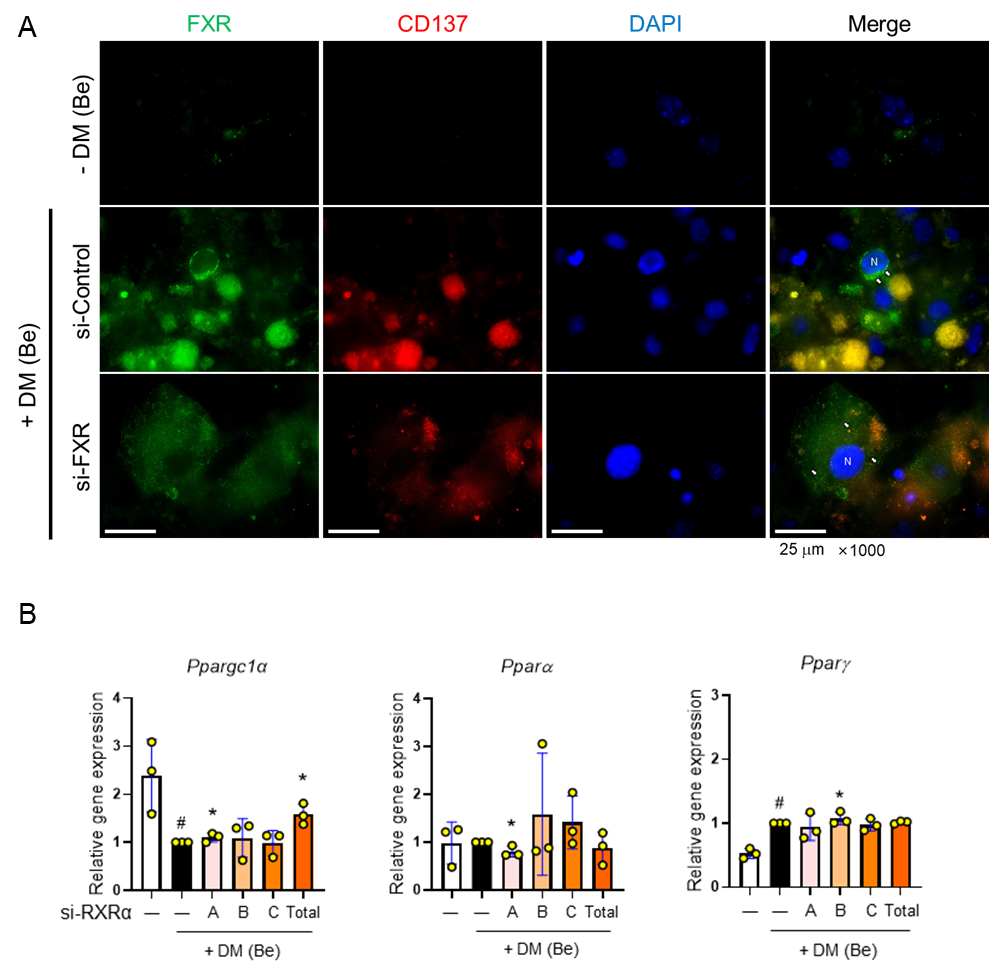
***

***Supplementary Figure 4***

(A) Expressions of FXR (green), CD137 (red), and nuclei (blue) were detected by immunofluorescence staining (magnification ×1000, scale bar = 75 µm) in beige-induced 3T3-L1 cells treated with si-FXR or si-Control. Arrows indicate cytoplasmic and nuclear localization of FXR expression in si-FXR and si-Control treated cells respectively. (B) The mRNA expressions of *Ppargc1α*, *Pparα*, and *Pparγ* were measured by RT-PCR in beige-induced 3T3-L1 cells treated with si-RXRα A-C isotypes or si-Control (20 nM). Total indicated three isotypes combination treatment as final concentration of 20 nM. All data are expressed as the mean ± SD of three or more independent experiments. Statistical differences were evaluated using an unpaired *t*-test and a subsequent *post hoc* one-tailed Mann-Whitney *U* test. ^#^*p* < 0.05 vs DM (Be)-untreated 3T3-L1 cells; ^*^*p* < 0.05 vs DM (Be)-stimulated 3T3-L1 cells. DM (Be), Beige adipocyte differentiation media; FA, farnesol.

***
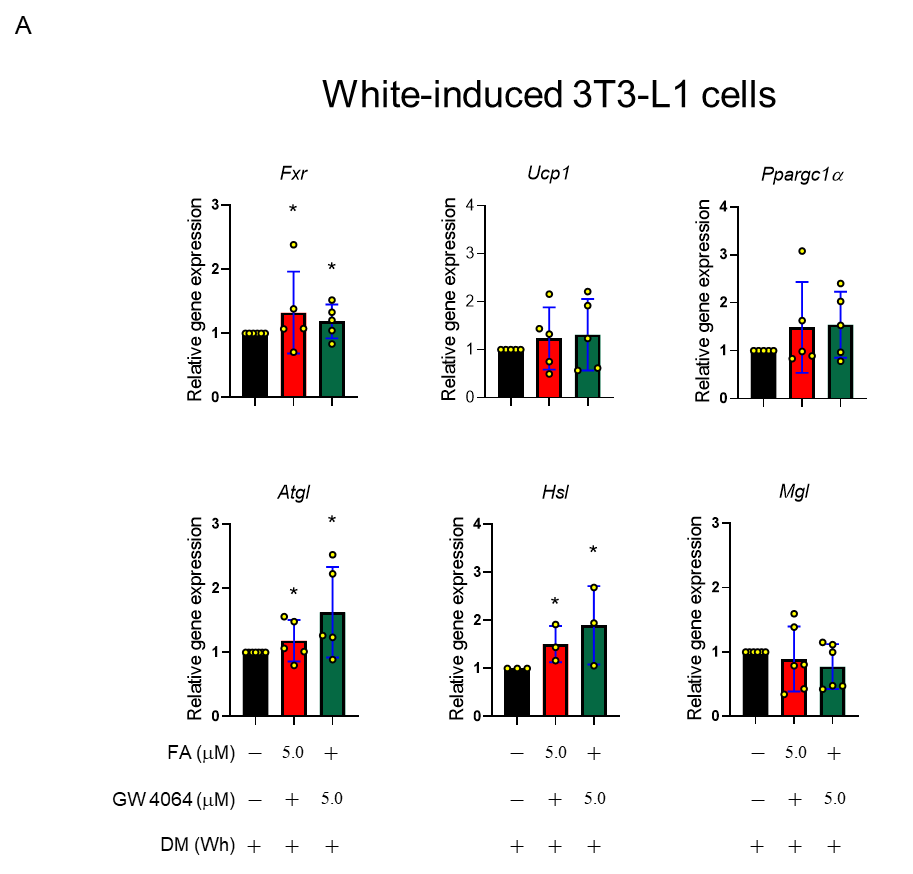
***

***Supplementary Figure 5***

(A) The mRNA expressions of *Fxr*, *Ucp1*, *Ppargc1α*, *Atgl*, *Hsl*, and *Mgl* were measured by RT-PCR in white-induced 3T3-L1 cells treated with Farnesol or GW4064. Results were expressed relative to *Gapdh*. All data are expressed as the mean ± SD of three or more independent experiments. Statistical differences were evaluated using an unpaired *t*-test and a subsequent *post hoc* one-tailed Mann-Whitney *U* test. ^*^*p* < 0.05 vs DM (Wh)-stimulated 3T3-L1 cells. DM (Wh), white adipocyte differentiation media; FA, farnesol


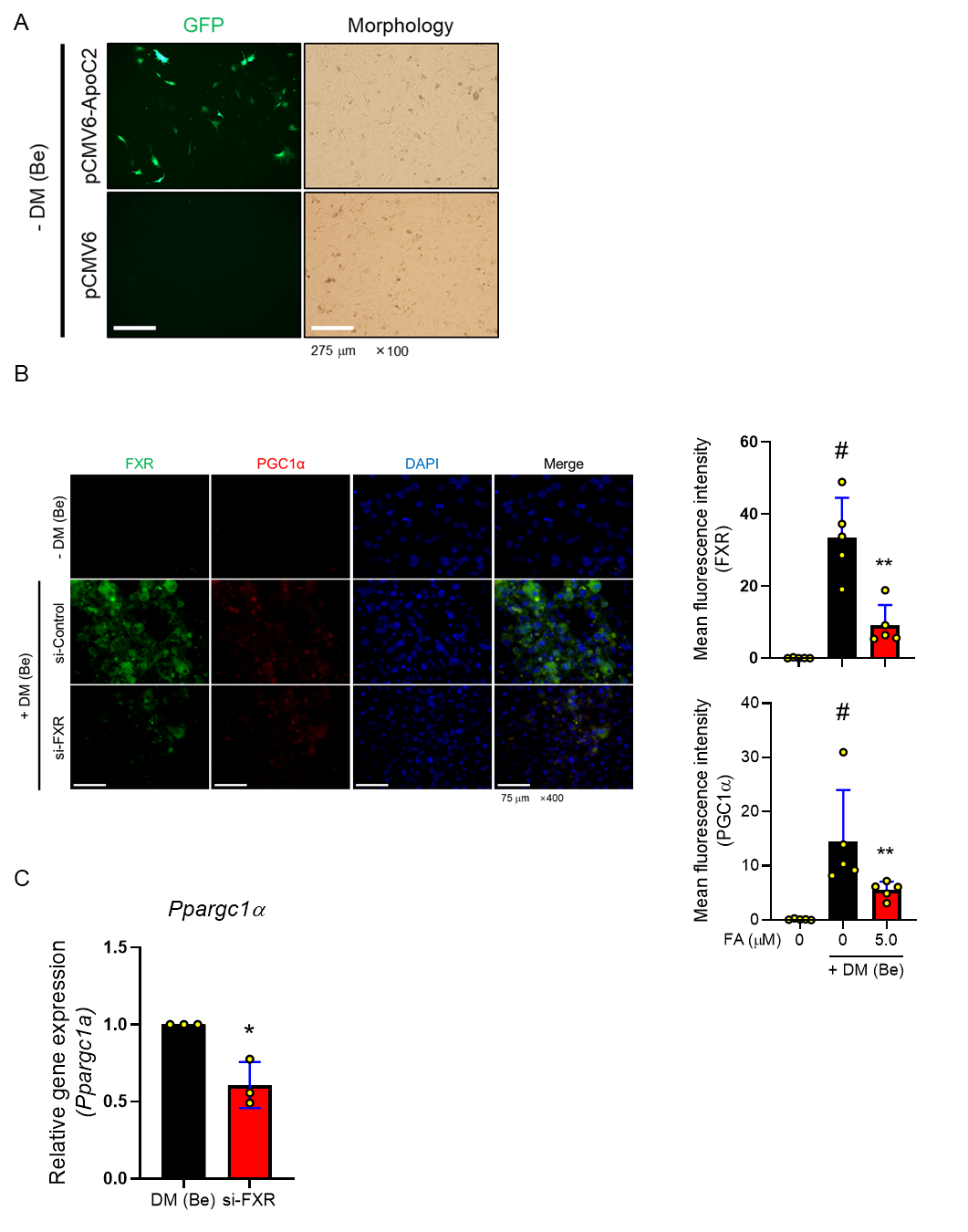


***Supplementary Figure 6***

(A) 3T3-L1 cells stably overexpress ApoC2. The bicistronic expression pCMV6-ApoC2 plasmid contained both the coding sequence of GFPs and an additional coding sequence for ApoC2. (B) Expressions of FXR (green), PGC1α (red), and nuclei (blue) were detected by immunofluorescence staining (magnification ×400, scale bar = 75 µm) in beige-induced 3T3-L1 cells treated with si-FXR or si-Control. Quantification of the fluorescence intensities was determined using ImageJ. (C) The mRNA expression of *Ppargc1α* was measured by RT-PCR in beige-induced 3T3-L1 cells treated with si-FXR or si-Control. Results were expressed relative to *Gapdh*. All data are expressed as the mean ± SD of three or more independent experiments. Statistical differences were evaluated using an unpaired *t*-test and a subsequent *post hoc* one-tailed Mann-Whitney *U* test. ^#^*p* < 0.05 vs DM (Be)-untreated 3T3-L1 cells; ^*^*p* < 0.05 vs DM (Be)-stimulated 3T3-L1 cells. *^**^p* < 0.01 vs DM (Be)-stimulated 3T3-L1 cells. DM (Be), Beige adipocyte differentiation media; FA, farnesol.

***Supplementary Table 1***

| **Category** | **Name of antibody** | **Company** | **Catalog Number** |
| --- | --- | --- | --- |
| Western blot 1^st^ Ab | UCP1 | GeneTex | GTX112784 |
|  | PPARδ |  | GTX113250 |
|  | TBX1 | Abcam | ab109313 |
|  | β3AR |  | ab-94506 |
|  | ATGL |  | EPR19650 |
|  | PPARγ | Cell Signaling Technology | 2435S |
|  | p-CREB (Ser133) |  | 9198s |
|  | CREB |  | 9197s |
|  | β-actin |  | 3700s |
|  | FXR | Santa Cruz Biotechnology | sc-25309 |
|  | TOM20 |  | sc-17764 |
|  | FXR | Thermo Fischer Scientific | PA5-40755 |
|  | PPARα |  | MA1-822 |
|  | PRDM16 |  | PA5-20872 |
| Western blot 2^st^ Ab | Goat anti-Mouse IgG |  | G-21040 |
|  | Goat anti-Rabbit IgG |  | G-21234 |
| Immunofluorescence staining 1^st^ Ab | CD137 | Abcam | ab203391 |
|  | TOM20 (F-10) | Santa Cruz Biotechnology | sc-17764 |
|  | UCP1 (A-6) |  | sc-518024 |
|  | FXR |  | sc-25309 |
|  | Histone H1 |  | sc-10806 |
|  | PGC1α | Thermo Fischer Scientific | PA5-38021 |
|  | DAPI |  | D1306 |
| Immunofluorescence staining 2^st^ Ab | Alexa Fluor 488 Goat anti-rabbit |  | A-11008 |
|  | Alexa Fluor 633 Goat anti-Mouse |  | A-21052 |

UCP1, uncoupling protein 1; PPARδ, peroxisome proliferator-activated receptor delta; TBX1, t-box protein 1; β3AR, β3 adrenergic receptor; ATGL, adipose triglyceride lipase; PPARγ, peroxisome proliferator-activated receptor gamma; p-CREB, phospho-cAMP-response-element-binding protein; FXR, farnesoid X receptor; TOM20, translocase of outer mitochondrial membrane 20; PPARα, peroxisome proliferator-activated receptor alpha; PRDM16, PR domain containing 16; PGC1α, peroxisome proliferator-activated receptor-gamma coactivator 1 alpha; DAPI, 4′,6-Diamidine-2′-phenylindole dihydrochloride.

***Supplementary Table 2***

| **Genes** | **Forward (5’ to 3’)** | **Reverse (5’ to 3’)** |
| --- | --- | --- |
| *m-Fxr* | GCT TGA TGT GCT ACA AAA GCT G | CGT GGT GAT GGT TGA ATG TCC |
| *m-Ucp1* | AGG CTT CCA GTA CCA TTA GGT | CTG AGT GAG GCA AAG CTG ATT T |
| *m-Atgl* | ATA TCC CAC TTT AGC TCC AAG G | CAA GTT GTC TGA AAT GCC GC |
| *m-Hsl* | CTG AGA TTG AGG TGC TGT CG | CAA GGG AGG TGA GAT GGT AAC |
| *m-Mgl* | ACC ATG CTG TGA TGC TCT CTG | CAA ACG CCT CGG GGA TAA CC |
| *m-Prdm16* | CCA CCA GCG AGG ACT TCA C | GGA GGA CTC TCG TAG CTC GAA |
| *m-Pparα* | AGA GCC CCA TCT GTC CTC TC | ACT GGT AGT CTG CAA AAC CAA A |
| *m-Pparγ* | TTT TCA AGG GTG CCA GTT TC | TTA TTC ATC AGG GAG GCC AG |
| *m-Pparδ* | TCC ATC GTC AAC AAA GAC GGG | ACT TGG GCT CAA TGA TGT CAC |
| *m-Cpt1α* | GAC TCC GCT CGC TCA TTC C | GAC TGT GAA CTG GAA GGC CA |
| *m-Cpt1β* | GCA CAC CAG GCA GTA GCT TT | CAG GAG TTG ATT CCA GAC AGG TA |
| *m-Ppargc1α* | AAT GCA GCG GTC TTA GCA | TGT TGA CAA ATG CTC TTC |
| *m-Rxrα* | ATG GAC ACC AAA CAT TTC CTG C | CCA GTG GAG AGC CGA TTC C |
| *m-Rxrβ* | GAA GGA CAC CGT TCC CCT TG | CCA GAG CGT AAG CTC AGC C |
| *m-ApoC2* | ATG GGG TCT CGG TTC TTC CT | GTC TTC TGG TAC AGG TCT TTG G |
| *m-ApoC2* | AGG ATA GTC CCT TCC TGC CA | TCC TTG GCA GAG GTC CAG TA |
| *m-ApoC3* | TAC AGG GCT ACA TGG AAC AAG C | CAG GGA TCT GAA GTG ATT GTC C |
| *m- Shp* | TGG GTC CCA AGG AGT ATG C | GCT CCA AGA CTT CAC ACA GTG |
| *m-Gapdh* | AAC TTT GGC ATT GTG GAA GG | GGA TGC AGG GAT GAT GTT CT |

Hsl, hormone-sensitive lipase; Mgl, monoacylglycerol lipase; Cpt1α, carnitine palmitoyltransferase 1 alpha; Cpt1β, carnitine palmitoyltransferase 1 beta; Rxrα, retinoid X receptor alpha; Rxrβ, retinoid X receptor beta; ApoC2, apolipoprotein C2; ApoC3, apolipoprotein C3; Shp, small heterodimer partner; Gapdh, glyceraldehyde-3-phosphate dehydrogenase.
